# Effect of Beveling Large Class II Cavities on the Enamel Marginal Quality of Direct Resin-Based Restorations

**DOI:** 10.3390/jcm14165649

**Published:** 2025-08-09

**Authors:** Andreas Rathke, Henry Frehse, Anne Selinka

**Affiliations:** 1 Faculty of Medicine, University of Ulm, 89081 Ulm, Germany; 2 Clinical Research, Dentsply Sirona, 78464 Konstanz, Germany; 3 Private Practice, 35037 Marburg, Germany

**Keywords:** adhesive, class II restoration, compomer, composite, enamel bevel, marginal quality, SEM

## Abstract

**Background/Objectives**: It is unclear whether enamel margins should be beveled in direct resin-based restorations. This study evaluated the influence of enamel beveling on the marginal quality of mesio-occluso-distal (mod) cavity boxes. **Methods**: Seventy-five caries-free human molars were divided into three groups. Mod-cavities with the entire margin in the enamel were prepared ± proximal bevel (*n* = 25). Twenty-five beveled mod-cavities served as control. Each group was restored with five material combinations: micro hybrid composite with etch-and-rinse (ER) or self-etch (SE) adhesive, compomer with ER or SE, and low-shrinkage composite with ER. A complex filling technique was used in the control. After artificial aging (1000 thermal cycles, 5/55 °C), the percentage of continuous margins (PCM) of the proximal boxes was analyzed by scanning electron microscopy using epoxy replicas (×300), and the marginal seal was assessed by light microscopy after dye penetration (×64). Statistical analysis was performed using Kruskal–Wallis and Mann–Whitney *U* tests (*p* < 0.05). **Results**: No significant differences in PCM were found between the restorative systems (*p* = 0.075). The composite with low shrinkage showed the highest mean PCM of all groups (78.1%). Significant differences in marginal seal were observed between the restoratives after bevel preparation (*p* < 0.05). Beveling significantly improved the PCM only for the hybrid composite (*p* < 0.05), whereby the effect on marginal seal was less pronounced. **Conclusions**: Clinicians should be aware that beveling mod-cavities does not necessarily improve the marginal quality of direct resin-based restorations bonded with well-established adhesives and may be more beneficial for traditional hybrid composites.

## 1. Introduction

Due to current legislation to phase-out amalgam, amalgam restorations are banned in several countries and are used less in others. Directly placed composite resin restorations have become a successful alternative to amalgam. Conventional micro and nano hybrid composites have demonstrated good aesthetic properties, adequate mechanical properties such as flexural strength and wear resistance, and high clinical survival rates [1,2]. However, polymerization shrinkage is still a critical limitation of direct composite restorations [3]. The shrinkage stress generated during polymerization is influenced by different factors such as the material properties of the adhesive and composite, the layering technique, the cavity preparation and their respective interactions [4,5]. The tensile stresses can cause negative effects at the adhesive interface, such as marginal gap, microleakage, fracture, postoperative hypersensitivity and secondary caries [1,2,3,4,5]. Secondary caries, especially at proximal margins, has been identified in clinical studies as the main cause of restoration failure [6,7].

The ability of adhesives to bond to enamel and dentin has opened up the possibility of defect-oriented preparations that no longer require a specific retention form, as is the case with amalgam or gold inlay restorations [8,9]. Successful bonding has been achieved with etch-and-rinse (ER) adhesives, which require separate phosphoric acid etching and rinsing steps to micromechanically interlock in enamel through resin tags and in dentin through a demineralized, collagen-rich hybrid layer. Alternatively, self-etch (SE) adhesives have simplified the bonding procedure by omitting the etching step through incorporation of acidic monomers that simultaneously etch and infiltrate enamel and dentin. In addition, dedicated acidic monomers can establish chemical (ionic) bonds with the calcium of the hydroxyapatite in the tooth [10]. However, the bond strength of SE adhesives to enamel was usually lower than those of ER adhesives and did not correlate with the acidity of the respective SE adhesive [11].

In the treatment of carious lesions, the focus has shifted to maximum tissue preservation [8]. The preparation suggested for adhesive restorations features a minimally invasive round or ovoid proximal box (box-only or slot cavity), including a 45° bevel at the enamel margins with a width of 0.5 to 1.0 mm [9]. The risk of iatrogenic damage to adjacent teeth is significantly reduced by beveling with oscillating instruments [12]. In small Class II composite restorations limited to the enamel, beveled proximal boxes showed less marginal gaps or microleakage than their box-shaped counterparts [12,13,14,15]. The improved adhesion was attributed to an accentuated etching pattern due to better prisms orientation, an enlarged adhesive surface, lower tensile stresses at the margins, and the removal of weakened or aprismatic enamel [9,16,17]. However, the cavities in these studies were generally smaller than the preparations required for the replacement of amalgam restorations [12,13,14,15,16,17].

Several working groups also have beveled enamel margins for larger Class II mod-restorations made of composite or compomer [18,19,20,21]. These cavities are subject to greater shrinkage stresses due to their cavity volume and have less enamel at the cervical margins to distribute stresses [4]. By extending the cavity with a bevel, the residual enamel is thinned out at the margins. However, as much enamel as possible should be preserved, especially at proximo-cervical margins, as bonding to enamel is more predictable in the long term than to dentin [10]. A recent study showed that enamel beveling does not improve the marginal quality of mod-composite restorations compared to box-shaped cavities [22]. The results suggested that the lower polymerization shrinkage of modern composites reduces or even eliminates any positive effect of beveling [22]. The authors concluded that the beveling of mod-cavities should no longer be recommended in composite restorations [22]. However, it is important for clinicians to back their clinical decision (i.e., the benefit-risk ratio of beveling) with more than one study. This is even more relevant as the clinical evidence on beveled versus box-shaped preparations is so far limited and contradictory [23,24].

Various other techniques have been suggested to mitigate polymerization shrinkage and stress. These include the use of complex filling techniques when placing the composite, alterations to the light-curing protocol, and modifications of composite formulations such as the use of low-shrinkage composite for bulk-filling or polyacid-modified composite (compomer) in pediatric dentistry [3,4,5]. The aim of this study was to investigate the effect of enamel beveling on the marginal quality of direct mod-restorations made of different composite types (hybrid composite, compomer, low-shrinkage composite). Since the effect of beveling is reported to also depend on the adhesive used [18,25,26], both ER and SE adhesives were selected. The null hypothesis was that the marginal quality of resin-based restorations is not influenced by the marginal design (box versus bevel) or the restorative system used.

## 2. Materials and Methods

**Figure 1:**
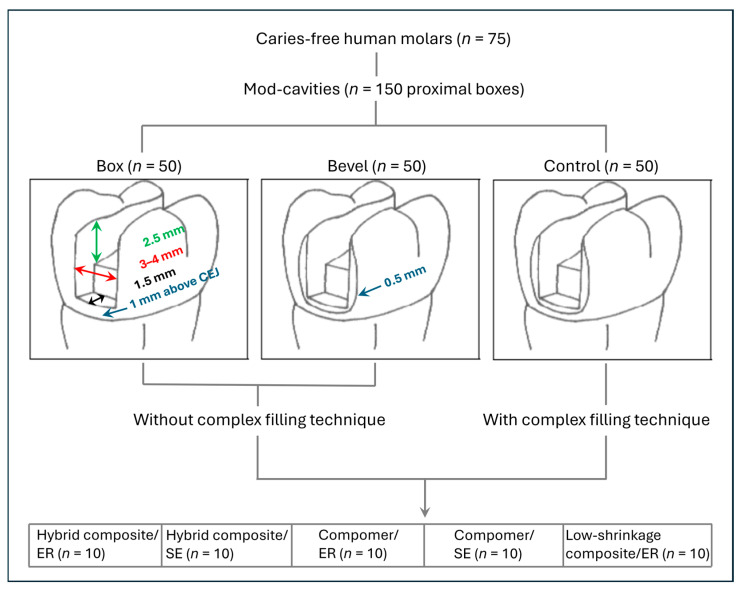
Group assignment and cavity dimensions under evaluation. CEJ: Cementoenamel junction; ER: Etch-and-rinse; SE: Self-etch.

For this study, 75 extracted human molars of comparable size and without caries were cleaned and stored in 1% chloramine T solution. The teeth were collected anonymously and could not be traced. All the donors provided informed consent for research purposes. In accordance with the principles outlined in the German ethics committee statement for the use of human body material in medical research [27], no ethical approval was mandatory for these samples and this type of study. Standardized mesio-occluso-distal (mod) box-shaped cavities with 2.5 mm-deep and 3.0 mm-wide occlusal boxes were prepared using 80 µm preparation diamonds (ISO No. 806 314 157524 012, Komet, Lemgo, Germany) in a high-speed handpiece (25 LH, Kavo, Biberach, Germany) under water spray. Proximal boxes were prepared with the same instruments to a bucco-lingual width of 4.0 mm and a depth at the bottom of 1.5 mm. The cervical margin of the proximal box was placed 1.0 mm above the cemento-enamel junction (Figure 1). The preparations were smoothened with 40 µm finishing diamonds (ISO No. 806 314 157514 012, Komet, Lemgo, Germany).

The prepared teeth were randomly divided into three groups of twenty-five teeth each (*n* = 50 proximal boxes). Box group: The proximal margins of the box-shaped cavities were not treated further. Bevel group: The proximal margins were beveled to 0.5 mm using 40 µm Bevelshape files (B40C, Intensiv, Montagnola, Switzerland) in a handpiece (INTRAmatic 20CN, Kavo, Biberach, Germany) with an oscillating head (INTRA Head 61 LR, Kavo, Biberach, Germany). Control group: The proximal margins were beveled as described. Each group was then randomly divided into five subgroups of five teeth (*n* = 10 proximal boxes), according to the combination of restorative materials and adhesives listed in Table 1.

The prepared teeth were placed in a customized dental model with adjustable adjacent teeth. Circular metal matrices (Kerr, Bioggio, Switzerland) were used in the box and bevel group for proximal contouring. Each matrix band was fixed in a Tofflemire retainer (Kerr, Bioggio, Switzerland) and placed interdentally cervically with wooden wedges (Hawe Sycamore Interdental Wedges, Kerr, Bioggio, Switzerland). The proximal box was restored with a micro hybrid composite or a compomer in one horizontal cervical layer and two oblique layers, while the low-shrinkage composite was applied in two horizontal increments. The remaining occlusal volume of the cavity was filled with two oblique layers (facial and lingual). Each layer of hybrid composite or compomer was light-cured for 40 s (irradiance 650 mW/cm^2^, Translux CL), while the low-shrinkage composite was light-cured incrementally for 10 s (high mode 1200 mW/cm^2^, Astralis 10). The control group was restored with transparent plastic matrices (Contact Molar, Ivoclar, Schaan, Liechtenstein) and wooden wedges (Hawe Sycamore Interdental Wedges) using a complex filling technique with three-sited light-curing [28]. Light-curing of the oblique facial and lingual layers in each proximal and occlusal box started through the facial and lingual walls for 20 s and ended occlusally for 30 s, while the oblique occlusal layer of each proximal box was light-cured occlusally for 50 s (Translux CL). After removal from the dental model, the restorations were finished and polished with flexible disks (Hawe Micro-Disc, Kerr, Bioggio, Switzerland) under water spray with loupes at ×2.5 magnification (Zeiss, Oberkochen, Germany). All cavity preparations and restorative procedures were performed by a single dentist (A.S.). Figure 1 summarizes the random assignment to the study groups.

For artificial aging, the restored teeth were thermocycled between 5 °C and 55 °C (1000 cycles, exposure time 30 s, Haake, Willytech, Munich, Germany). The artificial aging protocol was based on the ISO standard for testing adhesion to the tooth structure (test type 2) [29]. After thermocycling, impressions of the mesial and distal boxes were taken with a low-viscosity silicone impression material (Dimension Garant L, 3M, Seefeld, Germany) and filled with epoxy resin (Araldit, Ciba-Geigy, Basel, Switzerland). All epoxy replicas (*n* = 150) were glued with a carbon cement (Leit-C-Plast, Plano, Wetzlar, Germany) on aluminum specimen stubs (Agar Scientific, Rotherham, UK) and sputter-coated with gold in a sputtering device (Emitech K550, Röntgenanalytik Messtechnik, Taunusstein, Germany) at a conductive voltage of 25 mA for 2 min. The replicas were examined in the scanning electron microscope (SEM; Leica Stereoscan 420, LEO-Elektronenmikroskopie, Oberkochen, Germany) at approximately ×300 magnification by one trained operator who was blinded to the assignment of the specimens. SEM images were taken at an accelerating voltage of 15 kV with a working distance of 12–17 mm. The marginal quality was expressed as a percentage of the respective criterion (continuous margin, marginal gap and marginal fracture within the enamel or restoration) in relation to the assessable length of the proximal margin (100% reference). The occlusal restoration margin was not assessed.

For the microleakage test, the restored teeth were embedded in acrylic resin (Paladur, Kulzer, Hanau, Germany) and sealed with a waterproof varnish, leaving an area of approximately 0.5 mm from the restoration margins uncovered. The teeth were then immersed in 0.5% basic fuchsin solution at 37 °C for 24 h. After thorough rinsing under tap water, the teeth were sectioned longitudinally in the mesio-distal direction using a diamond saw (WOCO 50/Med, Conrad, Clausthal Zellerfeld, Germany) under water cooling. The depth of dye penetration along the resin–cavity interface and into the dentin tubules was recorded separately under a stereomicroscope at ×64 magnification (Stemi SV 8, Zeiss, Oberkochen, Germany).

Statistical analysis was performed using statistical software (IBM SPSS version 19 for Windows, IBM, Armonk, NY, USA). Since the Kolmogorov–Smirnov test indicated that the data from some groups were not normally distributed, Kruskal–Wallis and Mann–Whitney *U* tests were used to compare the groups. The significance level was set in advance at *α* = 0.05. Due to the exploratory nature of the research, raw *p*-values were reported and refrained from a correction for multiple testing.

## 3. Results

**Figure 2:**
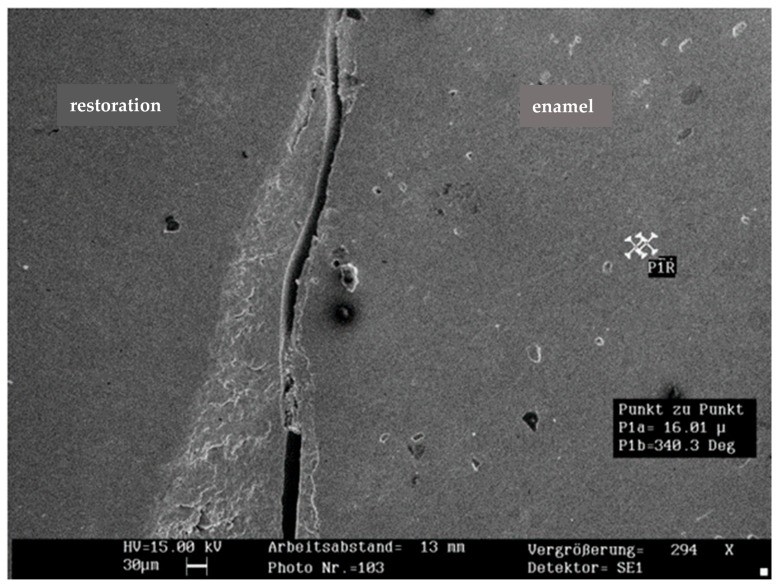
Representative SEM image of marginal fracture within the enamel (×294 magnification; 30 µm scalebar).

**Figure 3:**
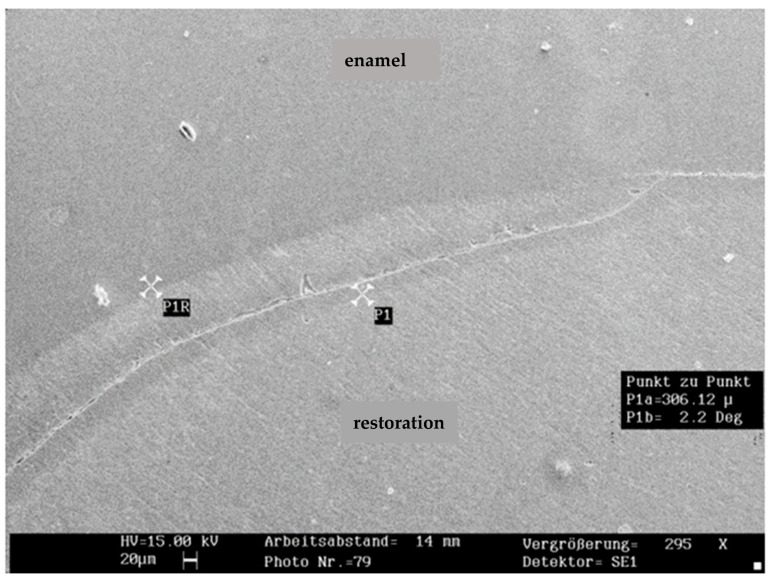
Representative SEM image of marginal fracture within the resin-based restoration (×295 magnification; 20 µm scalebar).

The mean percentage of marginal fractures among the groups was 1.2% in the enamel and 2.7% in the restoration (*n* = 150). Marginal fractures in the compomer (4.5%) were significantly more frequent than in the composite (1.6%; *p* < 0.05). Due to the low fracture incidence, no further analysis was performed. Representative SEM images of marginal fractures within the enamel and restoration are shown in Figure 2 and Figure 3.

**Figure 4:**
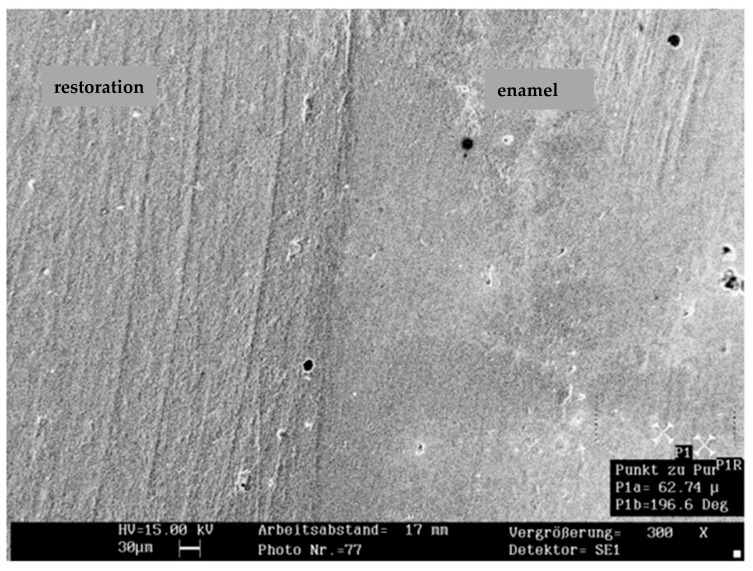
Representative SEM image of continuous margin (×300 magnification; 30 µm scalebar).

**Figure 5:**
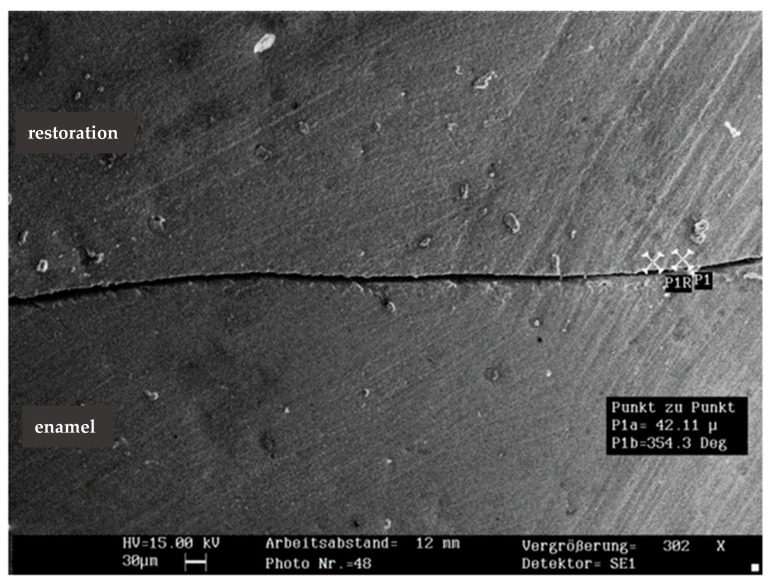
Representative SEM image of marginal gap (×302 magnification; 30 µm scalebar).

**Figure 6:**
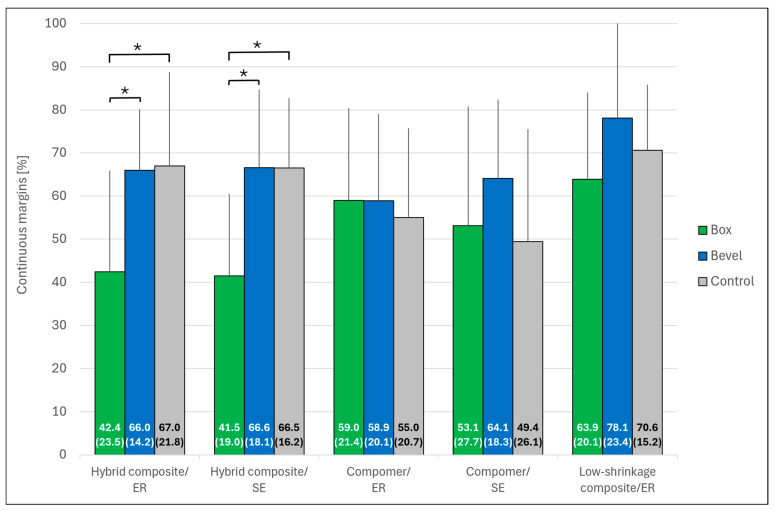
Percentages (mean ± SD; *n* = 10) of continuous margins, according to the marginal design and the different restorative systems. The mean percentage (SD) is given for each bar. Asterisks indicate significant differences within each restorative system group (*p* < 0.05). No significant difference was found between the restorative systems (*p* > 0.05). Control: Bevel plus complex filling technique; ER: Etch-and-rinse; SE: Self-etch. Error bars are only shown in the upper half for better visibility.

Figure 4 and Figure 5 show representative SEM images of the continuous margin compared to the marginal gap. Figure 6 presents the percentage of continuous margins (PCM) in all groups (the proportion of marginal gaps was approximately 100% minus PCM). The Kruskal–Wallis test revealed no significant differences in PCM between the five restorative systems (*p* = 0.075). The low-shrinkage composite bonded with ER adhesive provided the highest mean PCM in all three groups. Regardless of the adhesive tested (ER or SE), the hybrid composite showed the lowest mean PCM when the enamel margins were not beveled. Bevel preparation significantly improved the PCM for the hybrid composite with both adhesive and filling techniques (*p* < 0.05 in each case), while it did not increase the values of the other restorative systems (*p* > 0.05).

**Figure 7:**
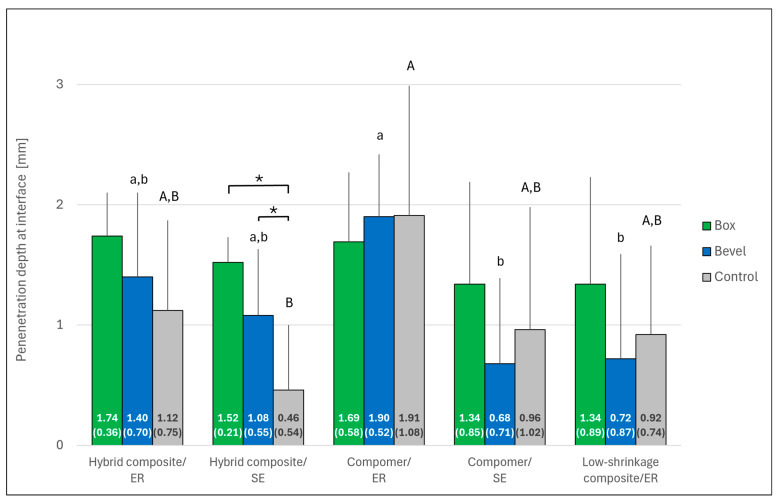
Depths (mean ± SD; *n* = 10) of dye penetration along the resin–cavity interface, according to the marginal design and the different restorative systems. The mean depth (SD) is given for each bar. Asterisks indicate significant differences within the restorative system group (*p* < 0.05). Restorative systems with the same small (bevel group) or capital (control group) letter are not significantly different (*p* > 0.05). No significant difference was found within the box group (*p* > 0.05). Control: Bevel plus complex filling technique; ER: Etch-and-rinse; SE: Self-etch. Error bars are only shown in the upper half for better visibility.

**Figure 8:**
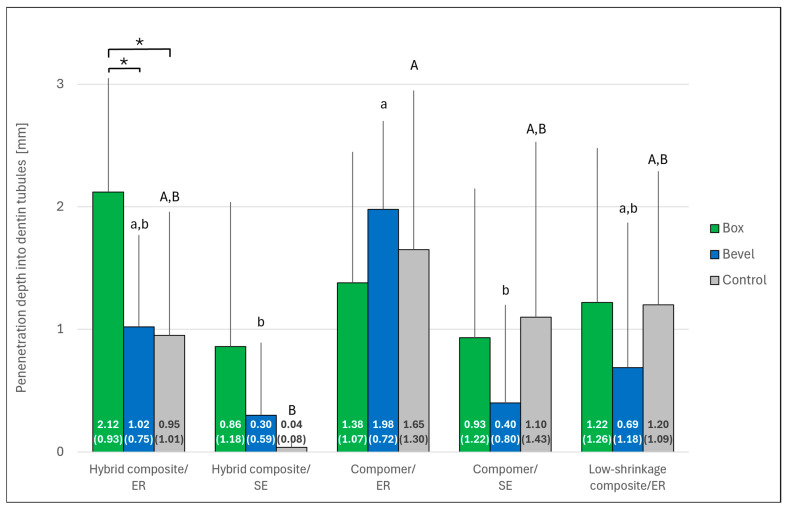
Depths (mean ± SD; *n* = 10) of dye penetration into the dentin tubules, according to the marginal design and the different restorative systems. The mean depth (SD) is given for each bar. Asterisks indicate significant differences within the restorative system group (*p* < 0.05). Restorative systems with the same small (bevel group) or capital (control group) letter are not significantly different (*p* > 0.05). No significant difference was found within the box group (*p* > 0.05). Control: Bevel plus complex filling technique; ER: Etch-and-rinse; SE: Self-etch. Error bars are only shown in the upper half for better visibility.

The results of dye penetration are shown in Figure 7 and Figure 8. Significant differences in penetration depth were observed between the restorative systems both along the resin–cavity interface and into the dentin tubules (*p* < 0.001 in each case). Post hoc testing confirmed significance between the groups after beveling (*p* < 0.05). In general, penetration depth was higher with ER than with SE, a trend that was significant for the compomer within the bevel group (*p* < 0.05). The effect of beveling was only significant for the hybrid composite bonded with ER adhesive (penetration depth into dentin tubules; *p* < 0.05).

## 4. Discussion

The present study showed that both the marginal design and the restorative system used have a significant influence on the marginal quality of direct resin-based restorations. Therefore, the null hypothesis had to be rejected.

In vitro tests (SEM analysis and fuchsin dye penetration) were performed to investigate the marginal quality of the restorations. SEM allowed the quantitative analysis of the restoration margin, but not the measurement of the gap depth. The microleakage test enabled the analysis of the penetration depth but required the specimens to be sectioned, which may lead to false interfacial leakage. The validity of the test has been questioned due to methodological limitations, high variability and low comparability of the results [29,30]. A correlation between dye penetration and the occurrence of marginal gaps could only be partially demonstrated [30]. Despite these reservations, the microleakage test was performed in this study because it has been used in comparable studies on the effect of enamel beveling [14,15,18,22,25,26].

While numerous study designs have beveled the enamel margins of direct composite or compomer restorations, especially in proximal boxes [12,13,14,15,18,19,20,21,25,26], other studies have found no positive effects of beveling [22,23]. The recommendation to bevel enamel margins is presumably based on results showing that transversely cut enamel prisms provide a better retentive etching pattern than longitudinally cut ones [9,16,31], thereby improving enamel bond strength [25,31] and marginal quality of the restorations [12,13,14,15,25,26]. The quality of the proximal margin is particularly important because interdental areas are less accessible to cleaning and more susceptible to secondary caries than the occlusal part of the restoration [6,7]. In the present study, proximal beveling did not improve the marginal quality of mod-restorations on a general basis. When comparing the different restorative systems, beveling significantly improved the percentage of continuous margins (PCM) only for the conventional micro hybrid composite, but showed no significant effect on the marginal quality (PCM and dye penetration depth) of compomer and low-shrinkage composite restorations.

Restorative materials and adhesives that have proven to be effective in clinical trials were selected for the study [32,33]. There were no significant differences in PCM between the restorative systems, with the low-shrinkage composite (precursor of Tetric EvoCeram Bulk Fill, Ivoclar, Schaan, Liechtenstein) showing the highest mean PCM values in all three groups. Clinical data confirmed these results. In combination with the same etch-and-rinse (ER) adhesive as used in this study, the low-shrinkage composite showed better clinical longevity in nonbeveled Class II cavities (mostly amalgam replacement cavities) after 15 years than a micro hybrid composite bonded with an ER adhesive from another manufacturer, although the difference was not significant [32]. The non-significantly higher PCM could be partly explained by the reduced polymerization shrinkage of the composite, which according to the manufacturer’s documentation is 1.6 vol%. The reduced shrinkage of the composite is obtained by reduction of the content of high-shrinkage dimethacrylates in the monomer and by use of a special copolymer filler resulting in a relatively high filler content of 81.9 wt% [32]. However, the marginal quality of the low-shrinkage composite could not be further optimized by beveling in the present study, regardless of whether a bulk-filling technique with metal matrices or a complex filling technique with three-sited light-curing and transparent matrices (control group) was used. It seems that the bonding potential of clinically well-established adhesives and the lower polymerization shrinkage of the composite compensated for the positive effect of enamel beveling [5,22].

The compomer restorations showed significantly more marginal fractures (4.5%) than the composite restorations (1.6%). Due to the somewhat lower mechanical stability of compomers in load-bearing Class II restorations [2], marginal beveling should be omitted to reduce the risk of marginal chippings/fractures in thin material layers. The results also showed that the compomer performed better than the hybrid composite in box-shaped cavities. The lower elastic modulus of the compomer compared to the micro hybrid composite may have contributed to the lower shrinkage stress during polymerization, thus reducing the tensile stress at the adhesive interface compared to the composite restorations [18]. In addition to the separate adhesive application, a certain degree of self-adhesion could be achieved in the compomer by the acidic resin monomer TCB (tetracarboxylic butane acid), which is a bi-ester of 2-hydroxyethyl methacrylate (HEMA) and butane tetracarboxylic acid. The monomer contains both two methacrylate and two carboxyl groups that polymerize with other monomers and form chemical (ionic) bonds with the calcium of the hydroxyapatite in enamel and dentin [34].

SEM analysis revealed no significant differences in PCM between the restorative systems and thus also between the adhesives used. However, some significant differences were found between the adhesives in the microleakage test suggesting that dye penetration was higher in the ER bonding mode than in the self-etch (SE) mode. Compared to SE adhesives which etch and prime simultaneously and are not rinsed off, the separate phosphoric acid etching and rinsing steps with ER adhesives could have caused disparities between the depths of enamel/dentin demineralization and subsequent resin infiltration into which the dye could penetrate. In addition, the better marginal seal of restorations bonded in the SE mode could be at least partly attributed to a chemical interaction potential of the contained acidic monomers with the enamel/dentin hydroxyapatite. However, the study did not investigate the ER and SE modes in current, trending universal adhesives [10]. Another limitation was that no undermining excavation was performed in the present cavities to better simulate clinical excavation of caries. In a microleakage study, additional excavation at the cervical margin had no significant influence on dye penetration in small Class II box-only composite restorations [14]. However, undermined enamel in larger mod-cavity boxes led to significantly more enamel fractures along the proximal margins of composite restorations, as revealed by SEM analysis [22]. The fracture incidence in this case (median value of 16.1%) was markedly higher than the percentage of enamel fractures in the present study (mean value of 1.2%). Whether undermined enamel margins should be removed, preserved (i.e., left in place), or built up adhesively with composite is the subject of current research [22,35]. Finally, the results of the present study need to be verified by further studies on this research question with other material combinations of adhesive/direct resin restorative and clinical investigations.

## 5. Clinical Relevance

Proximal beveling of mod-cavities did not improve the enamel marginal quality of compomer and low-shrinkage composite restorations bonded with clinically well-established adhesives and may be more beneficial for traditional micro hybrid composites. Clinicians should be aware that the risk of beveling large Class II cavities (loss of the already thin cervical enamel) seems to outweigh its benefit on the marginal quality of direct resin-based restorations.

## Figures and Tables

**Table 1:** Restorative systems and adhesive applications used in the study.

Restorative Material	Brand Name (Manufacturer; LOT No.)	Adhesive (Manufacturer; LOT No.)	Adhesive Application	Mode
Micro hybrid composite	Spectrum TPH (Dentsply Sirona, Konstanz, Germany; 0204000776)	Prime & Bond NT (Dentsply Sirona, Konstanz, Germany; 0204000566)	Apply etchant for 30 s (enamel) and 15 s (dentin). Rinse and gently air dry. Apply adhesive for 20 s. Gently air blow. Light cure for 20 s *	ER
		Adper Prompt L-Pop (3M, Seefeld, Germany; 132885)	Activate blister. Apply adhesive and rub for 15 s. Gently air blow. Light cure for 10 s *	SE
Compomer	Dyract Posterior (Dentsply Sirona, Konstanz, Germany; 0206001456)	Prime & Bond NT	Apply etchant for 30 s (enamel) and 15 s (dentin). Rinse and gently air dry. Apply adhesive for 20 s. Gently air blow. Light cure for 20 s *	ER
		Adper Prompt L-Pop	Activate blister, apply adhesive and rub for 15 s. Gently air blow. Light cure for 10 s *	SE
Low-shrinkage composite	InTen-S (Ivoclar, Schaan, Liechtenstein; E00484)	Excite (Ivoclar, Schaan, Liechtenstein; E51015)	Apply etchant for 30 s (enamel) and 15 s (dentin). Rinse and gently air dry. Apply adhesive and rub for 10 s. Gently air blow. Light cure for 10 s **	ER

Etchant (Manufacturer; LOT No.): 34.5% phosphoric acid (Vococid, Voco, Cuxhafen, Germany; 22838); ER: Etch-and-rinse; SE: Self-etch; * Irradiance 650 mW/cm^2^ (Translux CL, Kulzer, Hanau, Germany); ** High mode 1200 mW/cm^2^ (Astralis 10, Ivoclar, Schaan, Liechtenstein).

## Data Availability

Data are contained within the article.
